# Ingestion of supplements and fortified food with iodine on the breast milk iodine concentration in deficiency areas: a systematic review

**DOI:** 10.4314/ahs.v21i3.46

**Published:** 2021-09

**Authors:** Almeida Abudo Leite Machamba, Silvia Eloiza Priore, Mariana de Souza Macedo, Sylvia do Carmo Castro Franceschini

**Affiliations:** 1 Almeida A. L. Machamba: PhD Student in the Postgraduate Program in Nutrition Science, Department of Nutrition and Health, Federal University of Viçosa (UFV), Viçosa, Brazil; 2 Full PhD Professor in the the Postgraduate Program in Nutrition Science, Department of Nutrition and Health, Federal University of Viçosa (UFV), Viçosa, Brazil; 3 Post-Doctoral in the Postgraduate Program in Nutrition Science, Department of Nutrition and Health, Federal University of Viçosa (UFV), Viçosa, Brazil; 4 Full PhD Professor in the the Postgraduate Program in Nutrition Science, Department of Nutrition and Health and Pro-Rector of community affairs, Federal University of Viçosa (UFV), Viçosa, Brazil

**Keywords:** Iodine, supplements, fortified foods, breastmilk, iodine concentration

## Abstract

**Introduction:**

The level of iodine in breast milk may be inadequate and compromise the health of this, both due to excess and lack, some population groups remain deficient because of the low consumption of iodate salt, because there is an increase in consumption of other sources of iodine, such as supplements and fortified foods.

**Objective:**

To evaluate the effect of the consumption of fortified foods and nutritional supplements with iodine on maternal milk levels.

**Methodology:**

Systematic review based on the Prism method, using the descriptors provided by DeCS. The reading, selection and analysis of the methodological quality of the articles was done by two researchers independently.

**Results:**

From 346 abstracts, 6 were eligible. The median iodination range between the studies ranged from 75 to 600 µg in supplements and 150 and 225 µg in fortified foods with effect on increased iodine concentration of breastmilk (BMIC), achieving the adequacy of the median BMIC in 4 of the 6 studies.

**Conclusion:**

Iodine ingestion through supplements or fortified foods results in improved iodine levels in breast milk.

## Introduction

The iodine deficiency in the world primarily affects the maternal-infant group, such as lactating women consequently the group less studied^1^. This has serious consequences on women's health, but also impacts the child on breastfeeding, compromising neurocognitive and psychomotor development, and other neurological consequences^2^, ^3^.

The Breast milk is the primary food of the infant in the first 6 months of life, guaranteeing the availability of all the nutrients that the infant needs including iodine. However, the iodine intake by lactating women reflects the contribution of the infant in exclusive breastfeeding. Therefore iodine present in the milk, considered a good indicator of ingestion of this nutrient by the lactating women, because physiologically this in the organism, tends to concentrate more on breast milk, by recaptation of iodine present in the cytoplasm through the sodium iodine symporter and output of iodine in mammary gland 4.

To ensure the infant iodine needs, from 90 to 110 µg/day, by the ingestion of 0, 78L of breast milk 5, the lactating women need to ingest 250 µg/day of this micronutrient, through the consumption of foods, supplements and the iodized salt, 1, 6–8 to maintain their levels on the average of 146 µg/L of breastmilk. And in cases of insufficiency, additional consumption of 150 µg/day of iodine through supplements. This measure is adopted by most part countries through iodation programs and has resulted in positive effects on the reduction of all forms of disorders caused by the deficiency of this mineral 9, 10.

In the world, although some countries have achieved iodine adequate 11–13, or excessive levels of iodine consumption 14–21, specific population groups remain deficient or excessive at the same time that they present with low consumption of iodized salt 10, 13, 14, 22, 23, which leads to the hypothesis that this should be consumed in other food sources, such as fortified foods and iodine supplements. Thus, the levels of this micronutrient in breast milk may be inadequate and compromise the infant's health, both due to excess and lack 24.

As the recommendations for reducing salt consumption in many countries have been observed as protection measures against the occurrence of chronic diseases non-communicable. Thus, alternatively, the use of fortified foods or the supplementation has been verified many times associated with change of iodine status in population 11. On the other hand, there is little availability of studies evaluating the impact of consumption of fortified foods and iodine supplements on the variation of levels in breast milk in lactating women, which justifies this review. The objective is to evaluate the effect of the consumption of fortified foods and nutritional supplements with iodine on iodine levels in breast milk.

## Methodology

A systematic review was conducted, based on the Preferred Reporting Items for Systematic Reviews (PRISMA) 25 methodology to select articles. This systematic review sought to answer the following question “what`s the change of the level of iodine in breastmilk of lactating women who receive supplements or foods fortified with iodine?” The protocol of this study was registered in the PROSPERO with the identification number CRD42019122219.

The research was conducted from September 1st of 2018 to April 30st of 2019. To identify the articles, we conducted the search in MEDLINE (Pubmed), Science Direct, Scopus databases and Cochrane Library. Using the descriptors: “Iodine AND drugs OR supplementation AND breast milk iodine concentration AND urinary iodine concentration”, “Iodine AND iodine supplementation AND iodine fortification OR foods OR condiments OR dietary supplements OR food fortified OR food consumption AND breast milk iodine concentration AND urinary iodine concentration”: provided by DeCS (an acronym for Descriptores en Ciencias de la Salud: Health Sciences Descriptors) in English, Portuguese and Spanish, without filters.

For inclusion criteria original articles were included from randomized clinical studies, cohort and case-control that focus effect of maternal iodine intake, present in nutritional supplements and/or fortified foods in breastfeeding period on breast milk iodine concentratin. Studies were chosen if: i) participants received iodine supplements or fortified foods and ii) an appropriate control group was included which comprised participants who either received no supplements or fortified foods iii) all participants had to be breast milk iodine concentration with outcomes.

By two independent researchers the articles were selected after finishing elimination of duplicities by database and among databases.. In the case of divergencies, a third author was invited to include or discard. Information was extracted from the year, authorship, place of the study, type of design, target population, sample size, type, dose and duration of ingestion of the supplement and/or the fortified food, amount of iodine in the supplements, fortified food, breast milk and urine.

The quality of the studies were assessed according to the type of the study, therefore randomized clinical trials were assessment with Jadad et al. method 26. Studies were graded as high, moderate and low quality according to specific scores on the randomisation procedure, blinding of participants and investigators, and withdrawal rates from the study. Another studies (case-control and cohort) by Newcastle-Ottawa scale 27, 28. Points were awarded for each study as follows: maximum of 4 for study selection, 2 for comparability and 4 for exposure or outcome. Based on total scores, studies were graded as high (9–10 points), moderate (7–8 points) or low (<7 points) quality.

Although some articles were of low quality, because they were published in high-impact journals, the authors included in the study.

## Results

**Figure 1:**
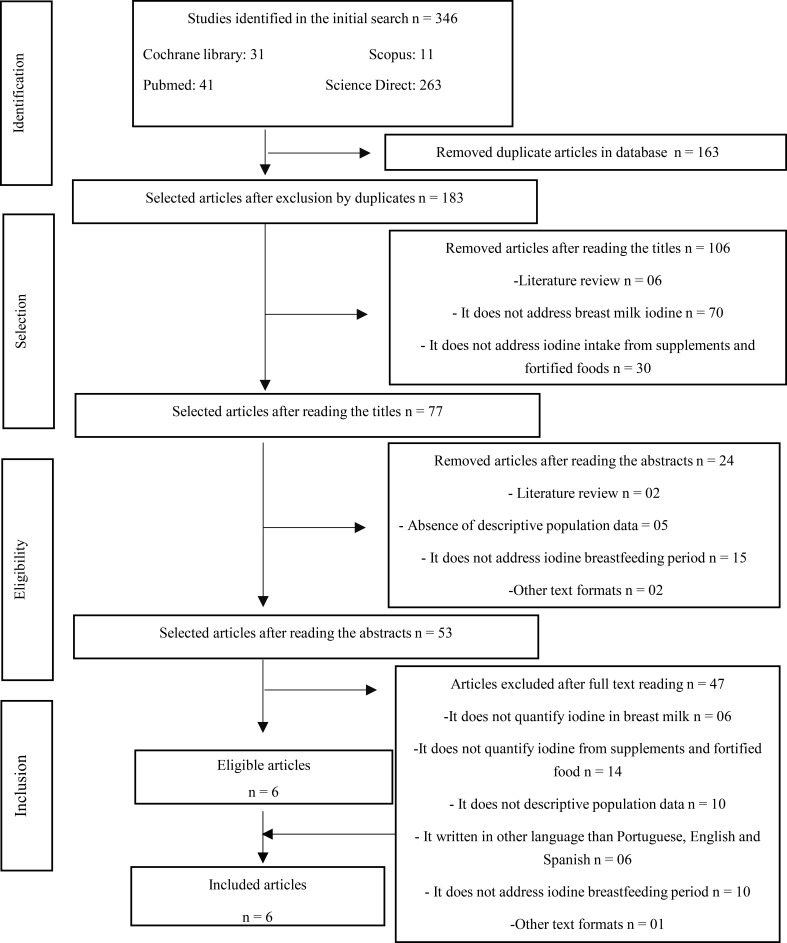
Identification and selection of articles.

The search resulted in 346 articles, after the elimination of duplicates, reading of titles, abstracts and full tests, 6 were selected (Figure 1). The studies were excluded because they failed to meet the inclusion criteria.

The studies were developed in countries of the African, European, American, Asian and Oceania continents and dated from 1999 to 2017. Regarding the design, three were randomized clinical trials 19, 27, 30, two cohort 10, 17 and one case-control 29 (Chart 1).

**Chart 1:** Characteristics of studies of the ingestion of supplementation and iodine fortification in lactating women

Place of estudy (reference)	Study design	n	Region and social status	Product/application	Iodine content	Iodine recommendation	Intervention time
Italy 29	Case-control	22	Urban >SEL	1 PerMamma Abbott©/day	116 µg KI	290 µg I/day 31	3 days
New Zealand 27	Randomized clinical trial	84	Urban	1 table/day	75–150 µg KIO3	150 µg I/day 32	168 days
United States 10	Prospective cohort	16	Urban	2 tablets/day + 2 pills/day	2x75 µg KI 2x225 µg KI	150 µg I/day 32	1 day
Australia 17	Prospective cohort	944	Urban	1 pill/day	150 µg KI	150 µg I/day	141 days
Ethiopia 19	Randomized clinical trial	101	Rural, > SEL	1 pill/day 10.3g of iodized salt/day	225 µg KI 30–40 µg KIO3	225–350 µg I/day 33	183 days
Iran 30	Randomized clinical trial	84	Urban	200mL of cow milk/day	150 µg KI	150 µg I/day 28	28 days

> **SEL**: High Socio-economic Level **PerMamma Abbott**©: Vitamin supplement used in Italy **KI**: Potassium Iodide **KIO3**: Potassium iodate *I*: Iodine **NA**: Not Available.

In this review, the samples number ranged from one 17 study with small sample size of 16 and other one with large of 994 27 lactating women, supplemented with capsules, tablets and nutritional solutions of potassium iodide (KI), with doses that ranged from 75 µg 10 a 600 µg 17 daily and food intake like bread 19 and milk, a part form iodized salt 10, 17, 19, that were fortified with KI and potassium iodate (KO3). All administrations start between the 1st to 183 days (Chart 1).

In all the studies, the effect of iodine intake for lactating women on breast milk levels and in your urine (Chart 2) were presented.

**Chart 2:** Change from breast milk iodine concentration (BMIC) and urine iodine concentration (UIC) of the lactating women who consumed iodine supplements and/or fortified foods

Place of estudy (reference)	Cut-off points	Interventions	% of changes from BMIC in µg/L	% of changes from UIC in µg/L
Italy 29	BMIC: 110 ± 40 µg/L	116 µg KI/day (n=10) Control (n=12)	↑ mean in 3 days of intervention, with more 50µg in KI group, achieving adequacy in the 2 groups, but at 90° day of measurement there were ↓ in 75.0% in KI group and in 59.3% in control *.	NA
New Zeland 27	UIC: 290 µg/L BMIC: >114 µg/L 34	75–150 µg KIO3/day (n=40) Control (n=44)	↑ median of 6.3% with 75 µg I/day up to 168° day* and 23.1% with 150 µg/day up to 28° day†, and ↓ up to 40% in Control†. Not adequated.	↑ in median at 168° day in 122,9% with 75µg I/day†, 68% with 150 µg I/day* and 70% in controlo†. Not adequated.
United States 10	BMIC: 110 µg/L 35	600 µg KI/day (n=16)	↑ median of 516.5% from 1^st^ to the 8^th^ hours of measurement, but at 678.0% at the peak reached in the 6^th^ hour†. Achieving adequacy.	↑ in median of 184.4% in the 8^th^ hour of measurement, achieving adequacy.
Australia 17	BMIC ≥100 µg/L^36^	150 µg KI/day + iodized bread (n= 588) 150 µg KI/day + bread (n= 136) Control, Iodized bread (n=65) Bread (n=155)	↑ in the median of 95.0% in the group† and 28.6% in Control†, and both adequate.	NA
Ethiopia 19	UIC >100 µg/L BMIC: 146 µg/L 34	225 µg KI/day (n=51) Control: 10,3g iodized salt/day (30–40 µg KIO3/g of salt) (n=50)	↓ in the median of 30.2% in the KI group and 29.3% in Control, and both becoming inadequate according to the authors, but had a value above 100 µg/L.	↑ in the median of 10.3% in teh KI group and in 5.3% in control, and both remain in adequate.
Iran 30	UIC: ≥100 µg/L ^4^ BMIC: 150–180 µg/L 37	200mL iodized cow milk (150 µg KI/day) (n=42) Control (n=42)	↑ in the median of 37.5% in the KI group *, achieving adequacy and ↓ in 25.6% in Control*, not achieving adequacy.	↑ in the median of 48.3% in the KI group*, achieving adequacy and ↓ in 57.2% in the control*, Not adequated.

±: Deviation BMIC: Breast Milk Iodine Concentration UIC: Urine Iodine Concentration ↑: High ↓: Low *: P<0,05

†: P=0,001 NA: Not Available, an UIC of the lactating woman.

Regarding the quality analysis of the studies, we observed that three 10, 19, 29 were classified as low quality, two 17, 30 moderate and one 27 high.

## Discussion

### Supplementation

The supplementation in lactating women results in the improvement of iodine levels in breast milk, regardless of the administered dose and duration of supplementation.

It was possible to observe that supplementation with, 600 µg KI in 1 day (P < 0.001) 10 and 150 µg KI/day in 28 days (P=0,001) 27, altered the levels of iodine in breast milk, improving the content and making them adequate.

The correction of low iodine levels in breast milk to adequate in lactating women who presented iodine insufficiency, showed that supplementation and consumption of fortified foods as an effective practice for restoring adequate levels of breast milk (chart 1 and 2).

However, supplementation with iodine values below 150 µg daily in moderately deficient lactating women did not result in a change 27, but when theses supplemented at 150 µg the deficiency status changed to mild^29^.

In a study developed in Italy, where it was offered to lactating women with iodine deficiency, for 3 days of hospital treatment, one PerMamma Abbott© pharmaceutical solution per day that contained 116 µg of iodine, having reached iodine adequacy in breast milk in third day but don't have change for control group. The authors showed a reduction of their iodine levels after 3 months, becoming inadequate, when compared to other lactating women who were also submitted to hospital treatment but who maintained only usual consumption of food fortified with iodine 29.

However, it is noticed that the supplemented dose was lower than the Food Drug Administration (FDA), which recommends that this should be done with 150 µg I/day for each nursing mother. Thus, it was understood that supplementation, with levels > 100 and < 150 µg I/day is successful in the acute treatment of deficiency for the quick replacement of iodine levels in breast milk, but for long-term maintenance, the consumption of fortified foods seems to be more effective^3^. On the other hand, in first days after deliver iodine supplementation or your intake for fortified foods not had impact in breast milk because in this time iodine breast milk concentration is stable^24^.

Still, supplementation with 150 µg I/day, is criticized by some authors, who claim that it is not enough to correct the severe or moderate deficiency of iodine in lactating women^38^. The Institute of Medicine of the United States (IOM) recommends that the best, is to practice a nutrient-rich diet that is balanced and varied diet over the days beyond only the use of supplements with iodine 39, 40, in the ingestion of iodine, it is important to have all the factors related to the ingestion of food and beverages that may influence the nutritional contribution of iodine in the lactating women 9, 10.

### Fortification

The consumption of iodine has been facilitated by the availability of fortified foods, as it is not available in the natural form.

It was possible to observe intake of 150 µg KI/day during 124 days, the fortified foods consumption such as iodized bread 17 and for 28 days the fortified milk (P < 0.05) 14 that change the levels of iodine in breast milk and making them adequate.

This strategy has been widely adopted as a measure of nutritional availability in iodine in many countries of the European, American and Oceania continents. So although salt consumption is recommended to adapt iodine levels, this has been related to the occurrence of non-communicable diseases (NCDs) 18, 41.

Newer studies have shown that the amount of salt ingested brings more risks related to NCDs, than benefits related to the supply of iodine 3, 18, 41. Reason why many European and Australia countries have been adopting programs to reduce salt ingestion in the population. Thus, the consumption of fortified foods with iodine, such as milk and its derivatives has been adopted in Europe and Asia 17, 42, bread in Oceania, Asia and Europe 10. This makes a measure of change of the food vehicle but also of the promotion of iodine consumption, as iodine is made available in other foods more pleasured and appreciated by the population. However, the monitoring of the iodine content to be supplemented in food is crucial.

### Clinical interpretation

Supplementation with high doses of iodine not only restores the iodine quickly but also corrects the deficiency, 38 even so it is recommended in severe to moderate iodine deficiency in lactating women with unavailability of foods rich in iodine in the diet 43.

According to FDA, KI should be used in the formulations of supplements and KIO3 in fortified foods, specifically to salt 3, 18, 41, however, in European countries such as Norway, Iceland and other Nordic countries, there are more than 20 formulations of dairy products fortified with iodine 9, 10. However, without any iodine reference approved by the FDA, which makes pertinent the existence of an international iodine reference and not only national for the fortification of food with iodine. Although, according to the Iodine Global Network, the type of iodine to be incorporated in food is not important, as well as the forms provide the amount of sufficient iodine that the body needs, although it is pointed out that the preference of the countries for using KI, is its low cost that is associated with its large production in the world 44. Meanwhile, the definition and approval of the vehicles to be incorporated is that it is the real concer, taking into account that each country has its eating habits.

The World Health Organization (WHO) indicates that the consumption of 250 µg of iodine per day by the lactating women is able to guarantee its concentration around 140 µg/L in breast milk, which keeps the iodine reserves for the infant. However, it was found that there are no reference values of iodine that classify the deficiency, adequacy or excessive amount in breast milk.

How alternative of salt reductions WHO recommends the consumption of 2 cups of milk (400ml) Daily, to provide 250 µg of iodine 45, In this study a cup of 200ml of milk per day was used to provide 150 µg of iodine, which makes the iodized milk a sufficient food source to satisfy the lactating women.

Although, as a policy of public health intervention, food fortification is the most effective and practical for the prevention of this deficiency 45.

However, the practice of supplementation should be considered in iodine insufficiency in lactating women, resulting from low coverage of adequate iodized salt in household, failed iodization programs, greater concern to reduce salt consumption or unavailability of food fortified with iodine beyond salt 46. Nevertheless, fortified foods emerge as an alternative to reduce salt consumption and increase iodine consumption, so people should be aware that its benefits are obtained in the medium and long-term.

The lack of an international reference value of iodine in fortified foods, besides salt in addition to the absence of an international reference for iodine in breast milk and its classification, which constituted a considerable limitation in this study and area of research, as it is difficult to discuss and compare data. On the other hand, by obtaining studies of at least one country from each continent, it allowed us to understand how the problem is global.

### Final Remarks

Supplementation results in improved iodine levels in breast milk. However, the iodine doses above 100 and below 150 µg/day are not adequate for iodine levels, but doses above 150 µg change the levels of deficiency. And this effect is the same when consuming fortified foods, and both (supplements and fortified foods) establish and remain in the body for long time.

In acute treatment in severe to moderate insufficiency conditions, supplementation with iodine doses of 600 µg nursing mother was effective in the daily iodine replacement in breast milk, which became adequate.

Further studies are required to verify the effect of supplementation and consumption of fortified foods with lower doses to restore iodine levels in breast milk as well as to establish cut off points for BMIC.
